# RNF8 promotes epithelial-mesenchymal transition of breast cancer cells

**DOI:** 10.1186/s13046-016-0363-6

**Published:** 2016-06-04

**Authors:** Jingyu Kuang, Li Li, Limei Guo, Yanrong Su, Yuxuan Wang, Yongjie Xu, Xiaozhen Wang, Shucong Meng, Liandi Lei, Luzheng Xu, Genze Shao

**Affiliations:** Department of Cell Biology, School of Basic Medical Sciences, Peking University Health Science Center, Beijing, 100191 China; Department of Pathology, School of Basic Medical Sciences, Peking University Health Science Center, Beijing, 100191 China; The Irma H. Russo, MD Breast Cancer Research Laboratory, Fox Chase Cancer Center-Temple University Health System, Philadelphia, PA 19111 USA; Department of Breast Surgery, the First Hospital of Jilin University, Changchun, 130021 China; Lab of Molecular Imaging, Health Science Analysis Center, Peking University, Beijing, 100191 China

**Keywords:** Breast cancer, Metastasis, EMT, RNF8

## Abstract

**Background:**

Epithelial-mesenchymal transition (EMT) is a crucial step for solid tumor progression and plays an important role in cancer invasion and metastasis. RNF8 is an ubiquitin E3 ligase with RING domain, and plays essential roles in DNA damage response and cell cycle regulation. However the role of RNF8 in the pathogenesis of breast cancer is still unclear.

**Methods:**

The expression of RNF8 was examined in different types of breast cell lines by Western Blotting. EMT associated markers were examined by Immunofluorescence and Western Blotting in MCF-7 when RNF8 was ectopically overexpressed, or in MDA-MB-231 when RNF8 was depleted. Transwell and wound healing assays were performed to assess the effect of RNF8 on cell mobility. The xenograft model was done with nude mice to investigate the role of RNF8 in tumor metastasis in vivo. Breast tissue arrays were used to examine the expression of RNF8 by immunohistochemistry. Kaplan-Meier survival analysis for the relationship between survival time and RNF8 signature in breast cancer was done with an online tool (http://kmplot.com/analysis/).

**Results:**

RNF8 is overexpressed in highly metastatic breast cancer cell lines. Overexpression of RNF8 in MCF-7 significantly promoted EMT phenotypes and facilitated cell migration. On the contrary, silencing of RNF8 in MDA-MB-231 induced MET phenotypes and inhibited cell migration. Furthermore, we proved that these metastatic behavior promoting effects of RNF8 in breast cancer was associated with the inactivation of GSK-3β and activation of β-catenin signaling. With nude mice xenograft model, we found that shRNA mediated-downregulation of RNF8 reduced tumor metastasis in vivo. In addition, we found that RNF8 expression was higher in malignant breast cancer than that of the paired normal breast tissues, and was positively correlated with lymph node metastases and poor survival time.

**Conclusions:**

RNF8 induces EMT in the breast cancer cells and promotes breast cancer metastasis, suggesting that RNF8 could be used as a potential therapeutic target for the prevention and treatment of breast cancer.

**Electronic supplementary material:**

The online version of this article (doi:10.1186/s13046-016-0363-6) contains supplementary material, which is available to authorized users.

## Background

Breast cancer (BC) is the most invasive form of cancer in women and is the second leading cause of cancer death in industrial nations [1]; the incidence rate of the disease has been rising in many countries [2]. Advanced breast cancer is associated with significant mortality because it metastasizes to vital organs. Tumor metastasis is a multistage process, among which epithelial-mesenchymal transition (EMT) is believed to be an initial step, during which non-motile, polarized epithelial cells lost their cell-cell junctions and converted into individual, non-polarized, motile and invasive mesenchymal cells [3, 4]. EMT can be induced or regulated by various growth and differentiation factors, including TGF-β, Wnt and Notch, as well as the tyrosine kinase receptor pathway [5, 6]. Activation of these pathways leads to transcriptional repression of a series of target genes that are involved in epithelial maintenance, including CDH1 gene, leading to the functional loss of E-Cadherin, one of the well-known hallmarks of EMT. The disappearance of E-Cadherin from adherent junctions results in the release of β-catenin into cytosol, and subsequent translocation to the nucleus where it can activate LEF/TCF (lymphoid enhancer factor/T cell factor)-mediated transcription, induce the expression of Snail, Slug and Twist [7], thereby contributing to the EMT program. Therefore, EMT is a cellular reprogrammed process that involves multiple layers of regulation for the gene transcription, including epigenetic regulation such as the post-transcriptional modification mediated by histone methylation and acetylation or deacetylation, as well as protein ubiquitination. It is assumed that many enzymes participated these processes are also important EMT regulators and play essential role in tumor metastasis [8, 9].

RNF8 is an ubiquitin E3 ligase with two conserved domains: the N-terminal FHA (Forkhead-Associated) and the C-terminal RING (Really Interesting New Gene) domain. The FHA can specifically bind phospho-peptides motif (pTXXF) in the target proteins [10, 11], while the RING is responsible for its E3 ligase activity. In response to DNA damage, gammaH2AX is phosphorylated, followed by the binding and subsequent phosphorylation of MDC1. This phosphorylation of MDC1 leads to the phosphorylation-dependent recruitment of RNF8 to the sites of DNA double strand breaks (DSBs), where RNF8 binds to the E2, Ubc13, and catalyses the formation of lysine 63-linked polyubiquitin chains (K63-Ubs) on histones [12–14]. The ubiquitination of histones at DSBs provides a structural chromatin platform for the subsequent recruitment of key DNA damage repair proteins such as 53BP1 and BRCA1, the latter of which is a well-known tumor suppressor, mutation of which strongly predisposes women to breast cancer [15–19]. In addition to the synthesis of K63Ubs, RNF8 can also catalyze the formation of K48-linked ubiquitin chains when coupled with other conjugating E2s such as UBCH8, UBE2E2, UbcH6, and UBE2E3 [20–24]. These RNF8-mediated K48-linked ubiquitin degradations play an important role in the control of protein abundance or turnover of many players, for example, JMJ2A and Ku80, which are involved in the DNA damage response (DDR) [22, 23].

In addition to its critical role in DDR, RNF8 is also implicated in many other biological processes, such as spermatogenesis, telomere end protection, mitosis and apoptosis [25, 26]. High-level amplification of RNF8 in lung cancer and leukaemia cells was revealed by the Cancer Genome Project (Sanger Institute) [27], but the role of RNF8 in tumorigenesis, especially in breast cancer metastasis remains poorly defined.

Here we report that RNF8 is overexpressed in highly metastatic breast cell lines and its overexpression can induce EMT in breast cancer cells. Furthermore, RNF8 is aberrantly expressed in invasive breast cancer and positively correlates with lymph node metastasis.

## Results

### RNF8 is overexpressed in malignant breast cancer cell lines

**Fig. 1:**
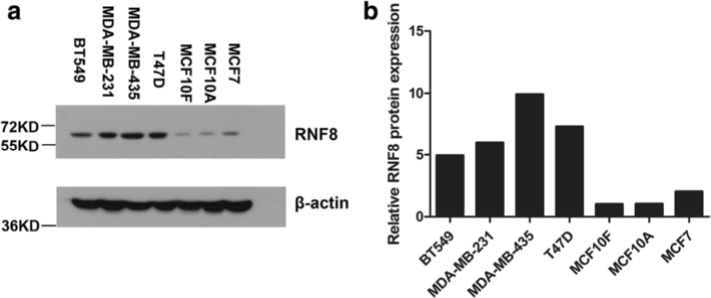
RNF8 is overexpressed in invasive breast cancer cell lines. **a** RNF8 protein expression in immortalized breast cell lines (MCF-10A and MCF-10 F), non-metastatic breast cancer cell lines (MCF7 and T47D), and metastatic breast cancer cell lines (MDA-MB-231, MDA-MB-435 and BT549), was detected by Western blotting. β-actin was used as an internal standard. **b** Quantitative analysis of the Western blotting bands shown in **a**

To determine the role of RNF8 in breast cancer, we first examined its expression in breast cell lines with various differentiated character by western blotting, including the immortalized breast cell lines MCF-10A and MCF-10 F, the epithelial breast cancer cell lines MCF7 and T47D, and the metastatic, mesenchymal breast cancer cell lines MDA-MB-231 and BT549, as well as MDA-MB-435, a breast metastasis-derived cell line of melanoma origin. Quantitative analysis of the Western blotting results was performed by normalizing the density of RNF8 band to that of β-actin. As shown in Fig. 1, the expression of RNF8 was increased in MCF7 (2.0-fold), and significantly elevated in T47D (7.3-fold), MDA-MB-231 (6.0-fold), BT549 (5.0-fold) and MDA-MB-435 (9.9-fold), as compared with the expression of RNF8 in MCF-10A and MCF-10 F, suggesting that the expression of RNF8 positively correlated with the metastatic potential of breast cancer cell lines.

### RNF8 induces EMT of breast cancer cell line

**Fig. 2:**
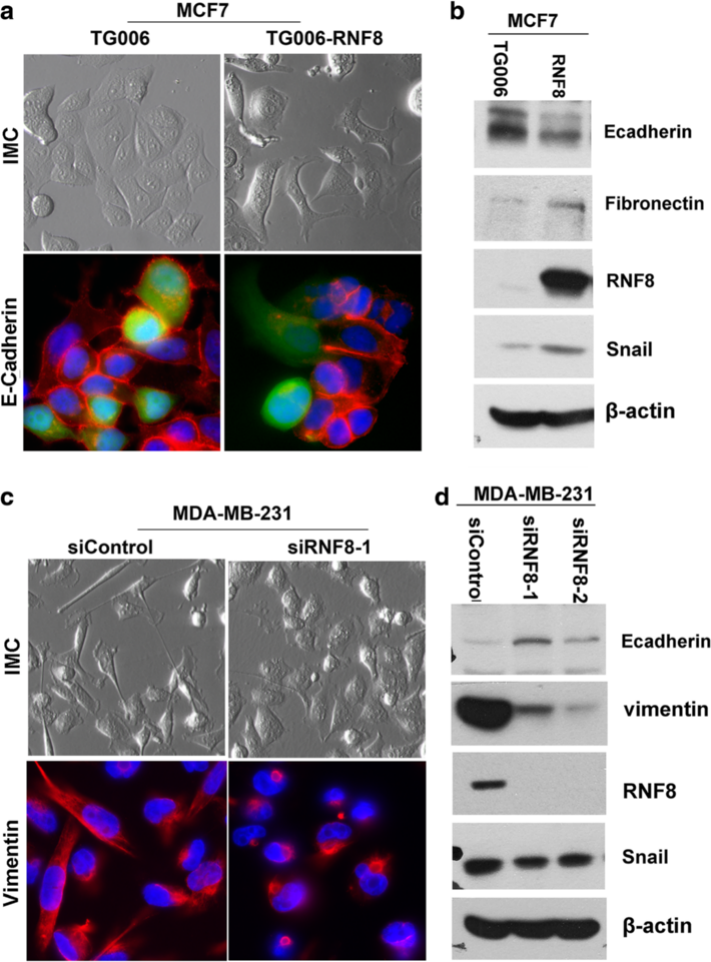
RNF8 regulates EMT in breast cancer cells. **a** Overexpression of RNF8 induced EMT in MCF7. MCF7 cells were infected with TG006 (empty vector with GFP) or TG006-RNF8 virus, the morphological change and the expression of E-Cadherin were examined 5 days later by IMC (*upper*) or by indirect immunofluorescence (*bottom*), using anti-E-cadherin antibody (*red*, E-cadherin; *blue*, DAPI). **b** Expression of the indicated EMT markers in the infected MCF-7 cells was detected by Western blotting. β-actin was used as an internal standard. **c** MDA-MB-231 cell lines were transiently transfected with 20nM siRNA (siControl or siRNF8-1), morphology of the transfected cells were examined 72 h later by IMC (*upper*), and the expression of mesenchymal marker was detected by indirect immunofluorescence (*bottom*) using anti-vimentin antibody (*red*, vimentin; *blue*, DAPI). **d** Expression of the indicated EMT markers in the infected MDA-MB-231 cells was detected by Western blotting. β-actin was used as an internal control. Magnification, 40 ×; bar: 5 μm

Epithelial-mesenchymal transition has been considered to be one of the critical steps involved in cancer metastasis [4, 6, 28]. Core elements of EMT include reduction of cell–cell adherence via the transcriptional repression and delocalization of cadherins, and functional loss of E-Cadherin is a well-known hallmark of EMT. Expression of epithelial intermediate filaments is typically reduced and the equivalent mesenchymal filament protein vimentin increased. To investigate what role, if any, RNF8 might play in the EMT, the morphological alterations and epithelial or mesenchymal marker changes in RNF8-expressing MCF-7 cells were assessed by microscopy and western blotting, respectively. As shown in Fig. 2a (upper panel), while control MCF-7 cells maintained organized cell–cell adhesion, overexpression of RNF8 in MCF-7 cells by lenti-viruses infection led to loss of cell–cell contacts; and these cells became scattering and gain a variable cell shape. In agreement with this, immunofluorescent microscopy showed a significant reduction or loss of E-cadherin staining in the cell membrane of RNF8-expressing MCF-7 cells compared with that of control cells (Fig. 2a, bottom panel). Consistently, overexpression of RNF8 in MCF-7 cells resulted in reduction of epithelial markers E-cadherin, and induction of mesenchymal marker fibronectin and snail (Fig. 2b). These results indicate that overexpression of RNF8 promotes EMT in MCF-7 cells. Whether depletion of RNF8 could induce MET, a reverse process of EMT, was also investigated in metastatic breast cancer cell line MDA-MB-231, using two individual small interference RNAs against RNF8 (siRNF8-1 and siRNF8-2). As shown in Fig. 2c, while control cells exhibited a typical spindle-like fibroblastic morphology, RNF8-depleted cells displayed a cobble stone-like appearance. Consistently, immunofluorescent microscopy showed a significant reduction in Vimentin staining in RNF8-depleted MDA-MB-231 cells as compared with that of the control cells (Fig. 2c, left, bottom panel). Immunoblotting showed that the protein level of Vimentin and snail was also decreased, while increased expression of E-caderhein, an epithelial marker, was detected in the cells depleted of RNF8 (Fig. 2d). Further examination of transcription of E-cadherin mRNA by quantitative real-time PCR (qPCR) showed that depletion of RNF8 in MDA-MB-231 cells indeed resulted in a dramatic increase in the transcription of E-caderhein gene (Additional file 1: Figure S1). Taken together, these results indicate that overexpression of RNF8 induce mesenchymal phenotypes, while depletion of RNF8 induce epithelial-like phenotypes of breast cancer cells, suggesting that RNF8 play an important role in EMT program.

### Overexpression of RNF8 inactivates GSK-3β and increases the accumulation of β-catenin

**Fig. 3:**
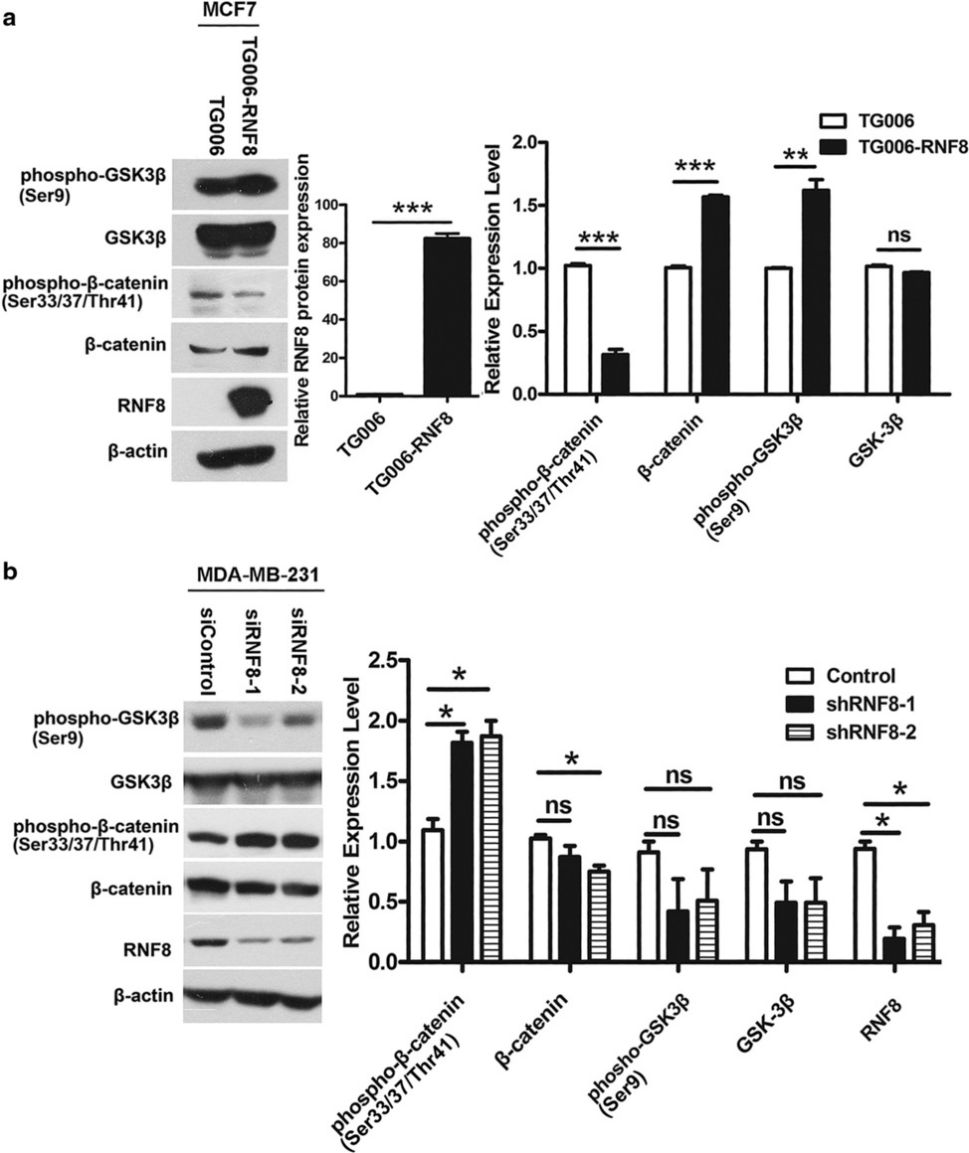
RNF8 inactivates GSK3β and increases β-catenin accumulation. **a** Western blotting analysis of EMT signaling hallmarks in MCF-7 cells infected with TG006 or TG006-RNF8 virus. β-actin was used as an internal standard. Quantification of each indicated protein expression was plotted (*right panel*). The result is shown from three independent experiments (Columns, mean; Bars, ± s.d. ***, *p* < 0.001; **, *p* < 0.01; *, *p* < 0.05; ns, no significance). **b** Western blotting analysis of EMT signaling hallmarks in MDA-MB-231 stable cell line expressed control (shRNA empty) or shRNF8-1 or shRNF8-2, β-actin was used as an internal standard. Quantification of each indicated protein expression was plotted in right panel (*right panel*). The result is shown from three independent experiments (Columns, mean; Bars, ± s.d. ***, *p* < 0.001; **, *p* < 0.01; *, *p* < 0.05; ns, no significance)

Glycogen synthase kinase-3 beta (GSK-3β) is a serine/threonine kinase, plays an important role in the initiation, progression and malignancy of many cancers [29, 30]. β-catenin is a crucial effector in EMT, which is phosphorylated by GSK-3β that targets it for ubiquitination and proteosomal degradation. Upon inhibition of GSK-3β, β-catenin accumulates in the cytoplasm and translocates into the nucleus, acting as a cofactor for the transcription factor LEF/TCF, affecting the transcription of genes that regulate EMT [31–33] including Snail. The activity of GSK-3β can be inhibited by activated Wnt-pathway or by PI3K/Akt-mediated phosphorylation at Ser9 of GSK-3β. We have found that the phospholation of GSK-3β (Ser9) is increased in RNF8-overexpressing MCF-7 cells. Consistently, the phospholation of β-catenin at Ser33/37/thr47, which is catalyzed by GSK-3β, is decreased, whereas the total β-catenin level is slightly increased in MCF-7 cells overexpressing RNF8 (Fig. 3a). In line with this, the phospholation of GSK-3β is decreased, while the phospholation of β-catenin is increased in RNF8-depleted MDA-MB-231 cells, stably expressing a small hairpin RNA against RNF8 (Fig. 3b). These data suggest that activation of β-catenin signaling is involved in RNF8 induced EMT.

### RNF8 enhances the cell migration potential of breast cancer cells

**Fig. 4:**
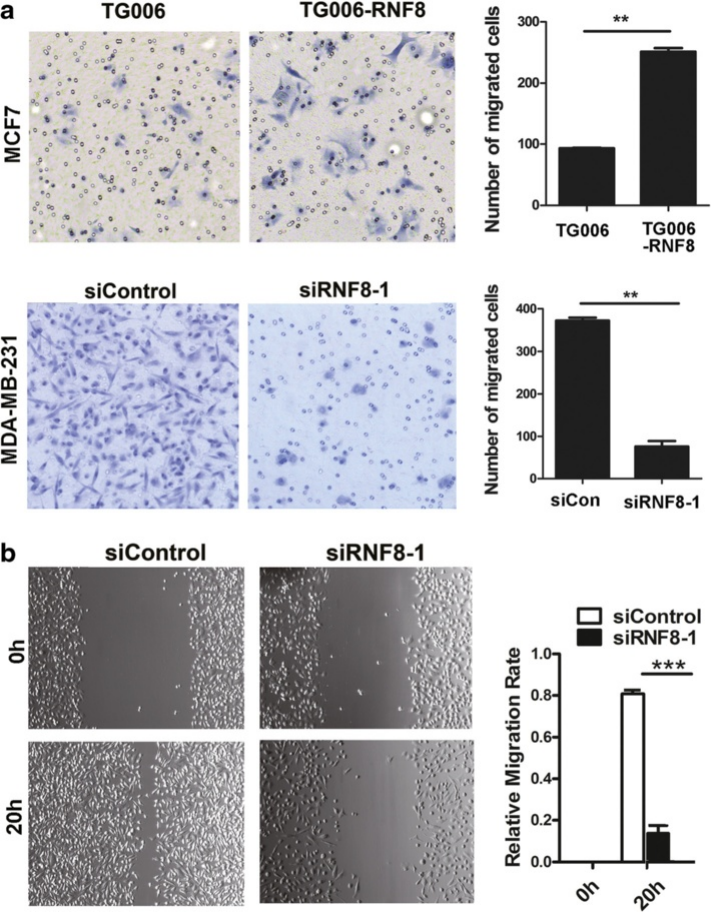
RNF8 promotes cell migration in breast cancer. **a** The migration of MCF-7 cells infected with TG006 or TG006-RNF8 virus (*upper*; magnification, 10 ×) or MDA-MB-231 cells transfected by siControl or siRNF8-1 (*bottom*; magnification, 20 ×) were examined by transwell migration assay. Overexpression of RNF8 enhances the migration of MCF-7 cells. **, *p* < 0.01. The data represent the means ± s.d. of three independent experiments. **b** Wound healing scratch assay was performed to detect the migration of RNF8-depleted MDA-MB-231 cells described in A. magnification, 10 ×; Right, the relative migration rate was calculated by dividing the change in the distance between the scratch edges by the initial distance, ***, *p* <0.001

Mesenchymal cells are associated with malignant properties, such as migration and invasion. To dissect the roles of RNF8 in EMT, RNF8 was either overexpressed in MCF-7 cells, or depleted in MDA-MB-231cells, and the role of RNF8 on the migration potential of these cells was investigated using transwell and wound healing assays. As shown in Fig. 4a, the cell migration potential was significantly elevated by 2.7 fold in RNF8-overexpressing MCF-7 cells (Fig. 4a, upper panel), while dramatically decreased to 1/4-1/6 fold of the control in RNF8-depleted MDA-MB-231 cells (Fig. 4a, bottom panel, and b). Taken together, these data demonstrate that RNF8 promotes cell migration in breast cancer cells.

### RNF8 promotes breast cancer metastasis in vivo

**Fig. 5:**
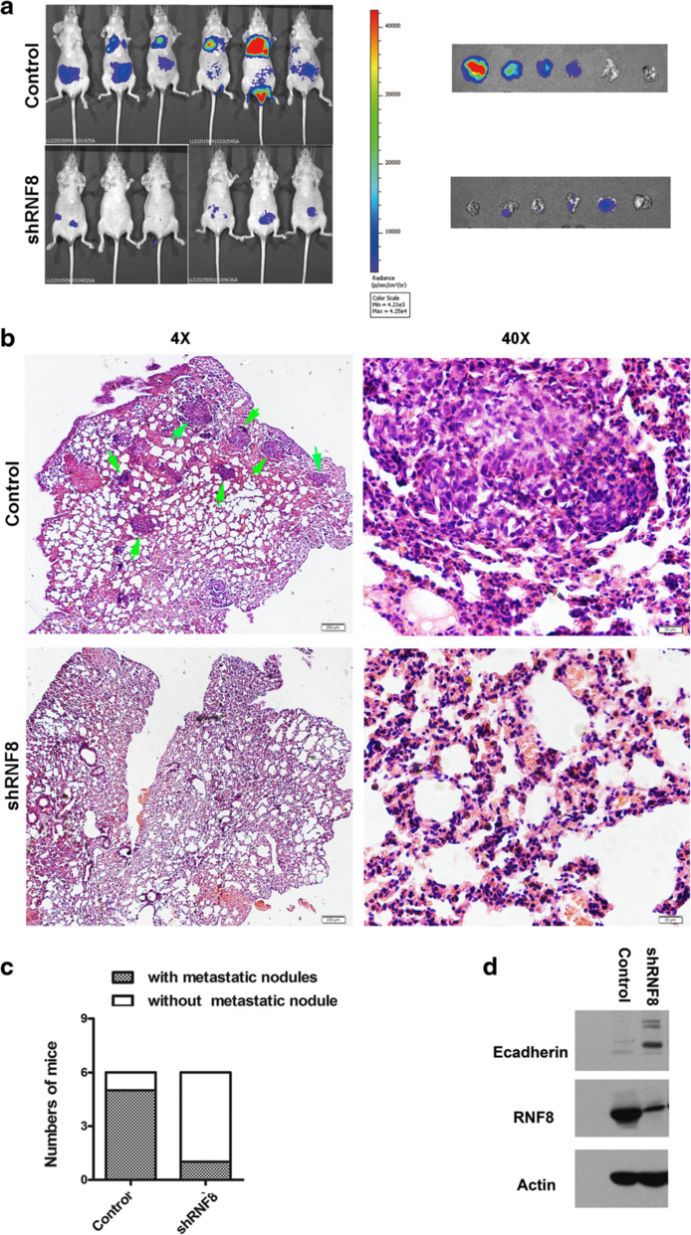
shRNA-mediated downregulation of RNF8 inhibits tumor metastasis in vivo. **a** Bioluminescence images in vivo. A total of 1 × 10^6^ /0.1 mL MDA-MB-231-luc cells stably expressing shRNF8-2 or an empty vector control were injected into caudal vein of 6–8 week-old female BALB/c mice (*n* = 6) (*left*), lung metastases were quantified using bioluminescence imaging. Lungs were harvested via necropsy 43 days after caudal vein injection and were examined by in vitro bioluminescent measurement (*right*). **b** Representative images of hematoxylin and eosin staining of lung sections. (magnification, 4 ×, left; 40 ×, *right*). Metastatic nodules were indicated by green arrows. **c** The number of mice with metastatic nodules in the lung sections. **d** Before injection, the expression of RNF8 and E-Cadherin in MDA-MB-231-luc-siCon and MDA-MB-231-luc-shRNF8-2 cells were examined by Western blotting

To further investigate the role of RNF8 in breast cancer metastasis in vivo, MDA-MB-231 cells that stably express firefly luciferase (MDA-MB-231-Luc-D3H2LN, Xenogen Corporation) were transfected with control or RNF8 shRNA to generate stable cell lines. The effect of RNF8 on tumor metastasis was assessed in immune-compromised female BALB/c mice (*n* = 6) by tail vein injection of MDA-MB-231-Luc-D3H2LN cells. The dissemination of tumors was monitored weekly by quantitative bioluminescence imaging with the IVIS imaging system (Xenogen), and a metastatic event was defined as any detectable luciferase signal above background. The results showed that the lung metastasis was dramatically decreased in mice implanted with MDA-MB-231-Luc-shRNF8 cells as compared with that of the MDA-MB-231-Luc-Control (Fig. 5a). Metastatic tumor foci in the lungs were confirmed by H&E staining. As shown in Fig. 5b, there are about 2–20 micrometastatic foci formed in each lung from the mice implanted with MDA-MB-231-Luc-Control cells; strikingly, only 0 or 5 micrometastatic foci was found in each lung from the mice injected with MDA-MB-231-Luc-shRNF8 cells. Moreover, HE staining also showed that the volume of metastatic foci seemed much smaller in animals injected with shRNF8 cells than that in control cells (Fig. 5b). Metastatic nodules in the lungs were found in five of the six control group mice (5/6), but only in one of the six shRNF8 group mice (1/6) (Fig. 5c); these data provided further evidence that RNF8 promotes breast cancer metastasis in vivo.

It should be noted that the expression of RNF8 and E-cadherin, as well as the migration potential in MDA-MB-231-Luc-shRNF8 and MDA-MB-231-Luc- Control cells were detected to select the positive clones (Fig. 5d). Before injection, the bioluminescence signals of MDA-MB-231-Luc-shRNF8 and MDA-MB-231-Luc-Control cells were also measured to make sure the cells used for implantation had the same luminescence intensity and activities (Additional file 1: Figure S2).

### RNF8 expression is associated with metastasis in human breast tumors

**Fig. 6:**
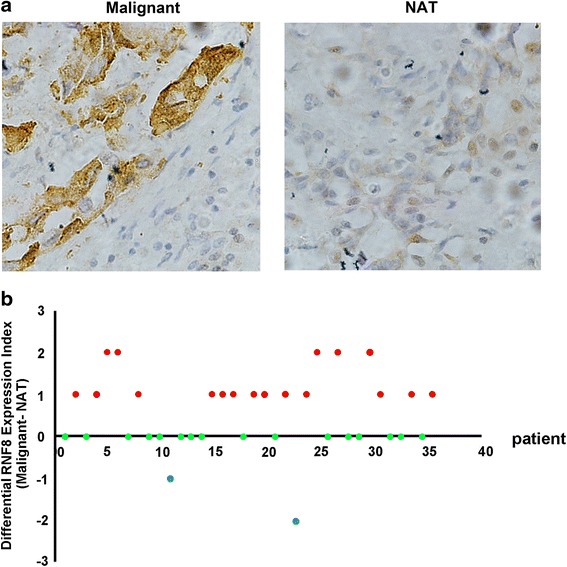
RNF8 is frequently upregulated in breast bancer tissues. **a** Expression of RNF8 was examined by IHC in primary breast cancers (Malignant) and the paired adjacent normal tissues (NAT). **b** RNF8 is differentially expressed in primary malignant breast cancer tissue compared with the corresponding paired adjacent normal tissue. For each case of the 36 patients, index of RNF8 expression (immunostaining score of IHC) of the primary tissue was subtracted by that of the corresponding paired adjacent normal tissue, and the resultant values (Malignant-NAT) from all 36 cases were used for plotting. The relative RNF8 expression level between primary cancer and paired adjacent tissue of each case was represented as a dot. RNF8 is considered to be significantly upregulated, similar or negative in primary tissue when compared with the corresponding paired adjacent tissue only if the calculated index is >0 (*red dots*), 0 (*green dot*), <0 (*blue dots*), respectively

To further support the role of RNF8 in promoting tumor metastasis, the expression of RNF8 was examined by IHC in breast cancer and the normal adjacent tissues, using tissue microarray. The histological diagnosis was made by pathologists according to the standard shown in Additional file 1: Table S1. The immunostaining pattern was divided into negative, low and high, according to the score based on total staining intensity and proportion of positive cells. The representative images were shown in Additional file 1: Figure S3. The expression of RNF8 was upregulated in breast cancers when compared with normal adjacent tissues (Fig. 6). Of the 36 cases examined, 18 (50.0 %) showed significant higher expression of RNF8 in the cancerous tissues when compared with the corresponding adjacent normal tissues; while another 16 (44.4 %) showed similar level of expression in both tissues; only 2 (5.6 %) displayed a reverse expression pattern, in which RNF8 expression was lower in the cancer tissues than that of normal adjacent ones. Statistical analysis showed a significant increased expression of RNF8 in cancer tissues (*P* = 0.006). This result indicates that RNF8 is aberrantly higher expressed in malignant breast cancer issues.

**Fig. 7:**
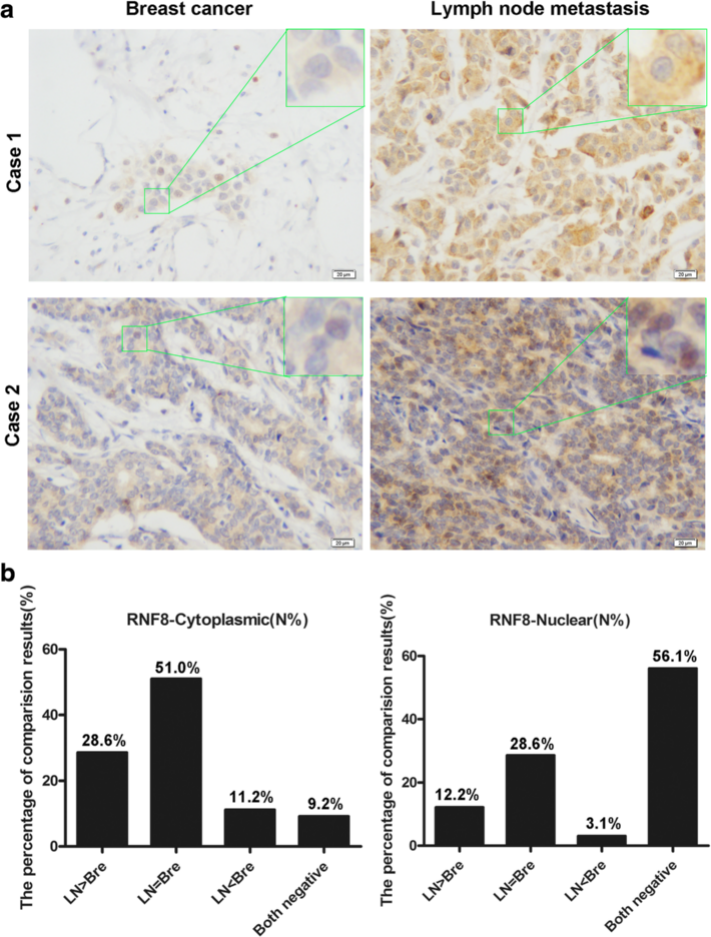
RNF8 expression is associated with breast cancer metastasis. **a** Representative examples showing higher RNF8 expression in the lymph node metastases than in the corresponding primary breast cancer tissues. case 1, cytoplasmic expression of RNF8; case 2, nuclear expression of RNF8. Magnification, 20 ×; scale bars: 20 μm; Inset, 40 ×. **b** Comparison of the RNF8 expression between breast cancer tissues and the corresponding lymph node metastases. LN > Bre, LN < Bre, and LN = Bre indicate RNF8 expression is higher, lower, or equal in metastatic lymph node compared with that of primary breast cancer; ‘Both negative’ means that the expression of RNF8 was not detectable in both metastatic lymph node and primary breast cancer tissues. The histographs show the percentage of cases with distinct expression pattern of cytoplasmic (*Left*) or nuclear (*right*) RNF8

To determine whether the up-regulation of RNF8 protein correlates with breast cancer metastasis, the expression of RNF8 was further detected by IHC in the primary cancer tissues and the paired metastatic lymph node. The histological diagnosis was made by pathologists according to the standard shown in Additional file 1: Table S1. The localization of RNF8 was depicted as cytoplasm or nuclear. The immunostaining pattern was divided into negative, low and high according to the score based on total staining intensity and proportion of positive cells; representative immunostaining pattern were shown in (Additional file 1: Figure S3). Of the 98 cases examined, 28 cases (28.6 %) showed higher cytoplasmic expression, 12 cases (12.2 %) showed higher nuclear expression in the lymph node metastases as compared with the corresponding primary cancers; the representative images were shown in Fig. 7a. However, another 50 cases (51.0 %) with cytoplasmic RNF8 expression and 28 cases (28.6 %) with nuclear RNF8 expression showed no significant difference between both tissues; a lower expression pattern in the lymph nodes was also observed in 11 of the cases (11.2 %) with cytoplasmic RNF8 expression, and 3 (3.1 %) cases with nuclear RNF8 expression, respectively; complete loss or negative expression of cytoplasmic or nuclear RNF8 was also observed in 9 (9.2 %) and 55 (56.1 %) cases, respectively (Fig. 7b). The expression level of RNF8 either in cytoplasm or in nuclear is significantly higher in lymph node metastases when compared with the corresponding primary cancers (*p* = 0.014, *p* = 0.016, respectively). These data suggest a positive correlation between expression of RNF8 and breast cancer metastasis.

We also analyzed the association between RNF8 expression and the clinic pathological parameters in breast cancer. As shown in Table 1, there was no significant correlation between the RNF8 expression levels and the parameters, including histological grade, clinical stage, and TNM.

**Table 1:** Association of RNF8 expression and clinical pathological features

Variables	N	RNF8-cytoplasmic N (%)			*P*	RNF8-Nuclear N (%)			*P*
		Negative	Low score	High score		Negative	Low score	High score	
		-	+	++		-	+	++	
Grade					0.658				0.659
1, 2	31	6 (19.4)	19 (61.3)	6 (19.4)		18 (58.1)	10 (32.3)	3 (9.7)	
3	49	9 (18.4)	34 (69.4)	6 (12.2)		29 (59.2)	20 (40.8)	0 (0)	
Stage					0.299				0.527
II (A + B)	62	13 (21.0)	40 (64.5)	9 (14.5)		35 (56.5)	26 (41.9)	1 (1.6)	
III (A + B + C)	33	3 (9.1)	25 (75.8)	5 (15.2)		22 (66.7)	8 (24.2)	3 (9.1)	
TNM-T					0.848				0.511
T1 + T2	83	16 (19.3)	54 (65.1)	13 (15.7)		50 (60.2)	30 (36.1)	3 (3.6)	
T3 + T4	14	2 (14.3)	11 (78.6)	1 (7.1)		10 (71.4)	3 (21.4)	1 (7.1)	
TNM-N					0.874				0.953
N0 + N1	69	13 (18.8)	47 (68.1)	9 (13.0)		44 (63.8)	24 (34.8)	1 (1.4)	
N2 + N3	27	5 (18.5)	18 (66.7)	4 (14.8)		18(66.7)	6 (22.2)	3 (11.1)	

**Fig. 8:**
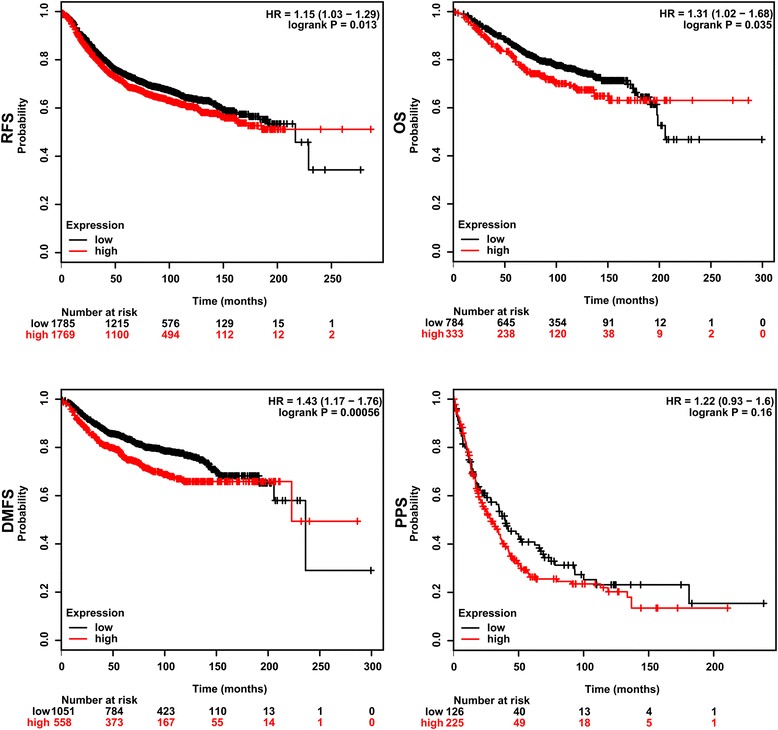
RNF8 expression reversely correlates with survival time. Kaplan-Meier survival analysis for the relationship between survival time and RNF8 signature in breast cancer was performed by using the online tool (http://kmplot.com/analysis/). A: RFS, Relapse Free Survival; B: OS, Overall Survival; C: DMFS, Distant Metastasis Free survival; D: PPS, Post Progression Survival. *P*-value < 0.05 was considered to be a statistically significant difference

To further extend our observations to a clinicopathologically relevant context, we performed Kaplan-Meier survival analysis of RNF8 with an online tool (http://kmplot.com/analysis/). The results showed that higher RNF8 expression was associated with a worse overall survival (OS, *P* = 0.035), worse Relapse Free Survival (RFS, *P* = 0.013), and worse Distant Metastasis Free survival (DMFS, *P* = 0.00056) of breast cancer patients, while there was no significant difference in Post Progression Survival (PPS) of breast cancer patients (*P* = 0.16) (Fig. 8), when the influence of systemic treatment, endocrine therapy, and chemotherapy was excluded. These data suggest that RNF8 could be used as a potential predictor of survival.

## Discussion

Epithelial-mesenchymal transition plays an important role in cancer invasion and metastasis, triggered by a diverse set of stimuli that can lead to stable reprogramming of epithelial cells to mesenchymal states. Here, we identify RNF8 as a novel factor involved in EMT and breast cancer metastasis. We have found that RNF8 is overexpressed in highly metastatic breast cell lines. Overexpression of RNF8 induces EMT in breast cancer cells, promotes breast cell migration and tumor metastasis in mouse xenograft model. Importantly, the expression of RNF8 is up-regulated in breast cancers tissues and is positively correlates with lymph node metastasis, establishing an important role for RNF8 in breast cancer metastasis.

RNF8 is an ubiquitin E3 ligase, localizes primarily to the nucleus during interphase; whereas under genotoxic stress (eg. IR treatment), RNF8 is localized to the sites of DNA damage, where it functions to ubiquitinate many chromatin substrates, including histone H2A, H2AX and H1 that are crucial for chromatin structure remodeling [11–14]. In addition, RNF8 also localizes at cytoplasm, especially upon some special stimuli (eg. stimulated by Human T lymphotropic virus type 1 (HTLV-1) trans-activator/oncoprotein, Tax), plays important roles in cell fate decision or genome stability through RNF8-mediated K-63 ubiquitination [34, 35]. In this study, we have found RNF8 protein expression is significantly higher in primary cancers than that of the matched noncancerous tissues. More importantly, a relatively larger proportion of either cytoplasmic or nuclear expression of RNF8 is higher in lymph node metastases than in the corresponding primary cancers. Overexpression of RNF8 led to the decrease of E-Cadherin, a hallmark of EMT, and RNF8 induced EMT is associated with the inactivation of GSK-3β and the accumulation of β-catenin. β-catenin is a critical player in the EMT signaling through shuttling from cytoplasm to nuclear, where it accumulates and complexes to TCF/LEF-1 to activate the downstream target genes involved in EMT [36–38]. These results reveal a strong association of RNF8 expression with EMT and breast cancer metastasis, raising the question on how RNF8 induces EMT signaling activation, and whether the increased expression in cancer cells is linked to an altered or disturbed RNF8 function, which in turn might contribute to the initiation and development of breast cancer. Further study will be needed to answer these questions.

Some metastasis-associated proteins are also important DNA damage response regulators, and hence contribute to IR/ anti-cancer drug resistance. One example is metastasis-associated protein 2 (MTA2), a subunit of the nucleosome-remodeling and histone deacetylation (NuRD) chromatin-remodeling complex. Depletion of MTA2 led to accumulation of spontaneous DNA damage and increased IR sensitivity [39]. RNF8 is one of the most important DNA damage repair molecules, playing crucial roles in DDR. Loss of RNF8 function can sensitize cells to both IR and DNA damage-inducing agents including anti-cancer drugs [11, 40]. One of the most difficult problems in the treatment of cancer is cancer metastasis and anti-drug resistance. Since RNF8 is aberrantly expressed in many breast cancer patients and facilitates tumor metastasis, and is a key player in the DDR pathway, it is therefore a promising target for anti-cancer therapy. By targeting RNF8, scientists could kill two birds with one stone in the future: not only the metastatic potential of cancer cells could be suppressed or eliminated, but also the efficacy of anti-cancer drugs could be significantly improved due to the increased sensitivity of cancer cells to anti-cancer drugs upon RNF8 depletion.

## Conclusions

In summary, RNF8 induces EMT in breast cancer cell line, and knock down of RNF8 reduces tumor metastasis in nude mice xenograft model. The expression of RNF8 is up-regulated in breast cancers tissues, positively correlates with lymph node metastasis and inversely correlates with survival time of the breast cancer patients. These findings highlight the potential of RNF8 as a therapeutic target of breast cancer.

## Methods

### Cell lines and culture

Human embryonic kidney HEK-293 T, Breast epithelial cell lines MCF-10A, MCF-10 F, MCF7, T47D and metastatic breast cancer cell lines MDA-MB-231, MDA-MB-435 and BT549 were purchased from American Type Culture Collection (ATCC) and preserved in our laboratory, MDA-MB-231-luc was a gift from Dr. Yongfeng Shang (Peking University Health Science Center). These cell lines were cultured in medium supplemented with 10 % FBS at 37 °C with 5 % CO_2_ in a humid atmosphere.

### Plasmid construction, siRNA and antibodies

TG006 is a lentiviral empty vector with GFP. For construction of TG006-hRNF8, full-length cDNA encoding RNF8 was amplified by PCR with Phusion DNA polymerase and subcloned into TG006. siRNA oligonucleotides (Invitrogen) were synthesized: siRNF8-1 (5′-GGACAAUUAUGGACAACAA-3′); siRNF8-2 (5′UGCGGAGUAUGAAUAUGAA-3′). Lentiviral vector shRNF8-1 (the same sequence with siRNF8-1: 5′-GGACAAUUAUGGACAACAA-3′, named as 26620), shRNF8-2 (5′-ACATGAAGCCGTTATGAAT-3′) and empty vector CONO77 were purchased from GeneChem. The following antibodies were used: mouse anti-RNF8 (sc-271462; Santa Cruz Biotechnology, Inc.); rabbit anti-E-cadherin (#3195, Cell Signaling Technology); rabbit anti-phospho-β-catenin (Ser33/37/thr47) (#9561, Cell Signaling Technology); rabbit anti-β-catenin (#8480, Cell Signaling Technology); rabbit anti-snail (#3879, Cell Signaling Technology); Monoclonal anti-Vimentin (V6389, sigma); rabbit anti-actin (sc-1616-R; Santa Cruz Biotechnology, Inc.); rabbit anti-phospho-GSK3β (Ser9) (#9336, Cell Signaling Technology); rabbit anti-GSK3β (#9315, Cell Signaling Technology).

### Transfection and infection

Lipofectamine RNAi MAX and Lipofectamine 2000 reagent (Invitrogen) were used for transient knockdown by siRNA or transient overexpression, respectively. All experiments were performed according to the manufacturer’s instructions. Lentiviral particles were produced by transfecting HEK293T cells with the TG006 vector or TG006-RNF8, as well as the psPAX2 and pMD2.G packaging vectors. 8 ug/ml polybrene was used for lentivirus infection of MCF7 cells and MDA-MB-231 cells.

### Protein extraction and western blotting

Immunoblotting was carried out to analysis the protein expression. Cells were lysed in RIPA buffer containing protease inhibitor cocktail and phospho-stop (Roche, Basel, Switzerland). Protein concentrations were determined using Bradford protein assays. Whole cell lysates were then fractionated using 8–10 % SDS–PAGE and electrotransferred onto polyvinylidene difluoride membranes (Millipore Corp., Bedford, MA, USA). The membranes were then probed with the indicated antibodies, and the immunoreactive bands were developed using an ECL detection system (Millipore, Billerica, MA, USA). All experiments were repeated for three times. For quantification, the grey density of the target bands in the immunoblot was analyzed by Image J software (National Institutes of Health, Bethesda, Maryland, U.S.) and normalized to the grey density of β-actin.

### Quantative realtime PCR

Total RNA was extracted using TRIzol reagent (Invitrogen) and cDNA was synthesized using SuperScript II Reverse Transcriptase (Invitrogen). Quantitative PCR was performed using an ABI PRISM 7000 Sequence Detection System (Applied Biosystems). E-cadherin amplification was performed using the following primers: 5′- TGGGCTGGACCGAGAGAGTTT-3′ and 5′-CGACGTTAGCCTCGTTCTCAG-3′. Samples were run in triplicates, and all samples were normalized against GAPDH using the comparative Ct method (△△Ct).

### Immunofluorescence microscopy

Cells grown on coverslips were fixed in 4 % paraformaldehyde at room temperature for 10 min, washed three times with PBS, blocked with 10 % goat serum at 37 °C for 30 min, incubated at 4 °C with the primary antibodies overnight, washed extensively and probed with the FITC- and Rhodamine red–conjugated goat anti–rabbit or anti–mouse IgG (Jackson ImmunoResearch Laboratories, Inc.) at 37 °C for 30 min. Coverslips were mounted in VECTASHIELD Mounting Medium with DAPI (Vector Laboratories).

### Cell migration and wound-healing scratch assay

Cell migration was measured according to the ability of the cells to migrate across a transwell filter (8-μm pores, Costar, Cambridge, MA, USA).1 × 10^5^ MDA-MB-231 cells or 2 × 10^5^ MCF7 cells suspended in serum-free DMEM were added to the upper chamber, and DMEM medium containing 10 % fetal bovine serum was added to the lower chamber. After a 20 h (for the siRNA-transfected cells) or 24 h (for the lentivirus-infected cells) incubation at 37 °C in a 5 % CO_2_ humidified atmosphere, the non-migrated cells were scraped off of the filter using a cotton swab and the cells that migrated to the lower side of the upper chamber, were fixed with 4 % paraformaldehyde and stained with hematoxylin. The cells per microscopic field (MDA-MB-231 cells, 20×; MCF7 cells, 10×) were taken pictures and counted in 8 randomly chosen fields. Triplicate wells were performed in each assay, and the assay was repeated at least three times.

One day before scratch, MDA-MB-231/RNAi cells were trypsinized and seeded equally into six-well tissue culture plates and grew to reach almost total confluence in 24 h. An artificial homogenous wound was created onto the monolayer with a sterile 10-ul tip. After scratching, the cells were washed with serum-free medium. Images of the cells migrating into the wound were captured at time points of 0 h and 20 h by inverted microscope (10×), all the experiments were repeated for at least 3 times.

### In vivo metastasis

MDA-MB-231 cells that stably express firefly luciferase (Xenogen) were infected with lentiviruses carrying control shRNA (CONO77), shRNF8-2, respectively. These cells were injected into the lateral tail vein (1 × 10^6^ cells) of 6–7 week-old female BALB/c mice (*n* = 6). For bioluminescence, IVIS Spectrum Imaging System (Xenogen, Alameda, CA, USA) was used. Lungs were fixed in formalin and embedded in paraffin blocks for slicing into thin sections. The paraffinized sections were stained with hematoxylin and eosin (H&E) according to standard protocols. The stained sections were photographed using a Leica microscope (Leica, Wetzlar, Germany).

### Immunohistochemistry (IHC) with breast tissue arrays

All breast tissue arrays were purchased from www.alenabio.com. BR804a with paired cancer adjacent normal breast tissue (40 cases/80cores, 4 cases missing), BR20837 included information on TNM (tumor, lymph node, metastasis) classification, clinical stage and histological grade (104 cases/208cores, 8 cases missing). The protocols of immunohistochemistry staining were also downloaded from www.alenabio.com. Antigen retrieval was performed by microwave oven method in Tris-EDTA-based solution, pH 9.0 to about 95 °C, 20 min. Blocking solution was used to prevent nonspecific binding of antibodies. The sections were incubated with mouse monoclonal anti-RNF8 antibody (Santa Cruz, sc-271462, 1:40 dilution) overnight at 4 °C. GTVision TM III Detection System/Mo&Rb (GK500705) was used for detection.

### Statistical analysis

The histological diagnosis was made by two pathologists. SPSS version 17.0 was used for statistical analysis. Comparisons between cancers and adjacent normal tissues were performed using two-sample paired wilcoxon signed rank test. Association of RNF8 with clinic pathologic factors using K-independent samples Cruskal-Wallis test. The differences between two independent groups were analysed using Student’s *t* test. *P*-value < 0.05 was considered to be a statistically significant difference. Kaplan-Meier survival analysis for the relationship between survival time and RNF8 signature in breast cancer was performed using the online tool (http://kmplot.com/analysis/).

## Abbreviations

BC, Breast cancer; DAPI, diamidino-phenyl-indole; EMT, epithelial to mesenchymal transition; H&E, Hematoxylin and eosin; IF, immunofluorescence; IHC, Immunohistochemistry; IMC, image motion compensation; IVIS, In Vivo Imaging Systems; qPCR, Quantitative Realtime PCR; RNF8, ring finger protein 8; siRNAs, small interfering RNAs.

## Additional file

Additional file 1:
**Table S1**. RNF8 Immunostaining Pattern Scoring. **Figure S1.** Real-time PCR analysis of relative E-cadherin mRNA change in RNF8-knockdowned MDA-MB-231 cell line. mRNA of GAPDH was used as a control. Error bars represent mean ± s.d. from three independent experiments; ***p* < 0.01. **Figure S2.** Bioluminescence signals of the cells. Before inoculated into the mice, the bioluminescence signals of MDA-MB-231-Luc-siCon or MDA-MB-231-Luc-shRNF8-2 cells were examined by bioluminescence imaging. **Figure S3.** Representative immunostaining pattern in the breast cancer tissues. Images show negative, low and high RNF8 expression respectively. (DOC 3980 kb)
