# Supplementary material for: RNF8 promotes epithelial-mesenchymal transition of breast cancer cells
Source: J Exp Clin Cancer Res. 2016 Jun 4;35:88. doi: 10.1186/s13046-016-0363-6 (PMC4893263; doi:10.1186/s13046-016-0363-6)
Supplement: Additional file 1: — Table S1. RNF8 Immunostaining Pattern Scoring. Figure S1. Real-time PCR analysis of relative E-cadherin mRNA change in RNF8-knockdowned MDA-MB-231 cell line. mRNA of GAPDH was used as a control. Error bars represent mean ± s.d. from three independent experiments; **p < 0.01. Figure S2. Bioluminescence signals of the cells. Before inoculated into the mice, the bioluminescence signals of MDA-MB-231-Luc-siCon or MDA-MB-231-Luc-shRNF8-2 cells were examined by bioluminescence imaging. Figure S3. Representative immunostaining pattern in the breast cancer tissues. Images show negative, low and high RNF8 expression respectively. (DOC 3980 kb) [file 13046_2016_363_MOESM1_ESM.doc]

**Supplemental information**

**Table S1 : RNF8 Immunostaining Pattern Scoring**

| **Staining Pattern** | **Score** |
| --- | --- |
| **Intensity** |  |
| Negative | 0 |
| Weak | 1 |
| Moderate | 2 |
| Strong | 3 |
| **Proportion of cells staining** |  |
| No positive cells | 0 |
| 1%-30% positive cells  30%-70% positive cells | 1  2 |
| 70%-100% positive cells | 3 |
| **Total score** | |
| Total score of 0, negative; 2~3, low expression +; 4~6, high expression ++ | |


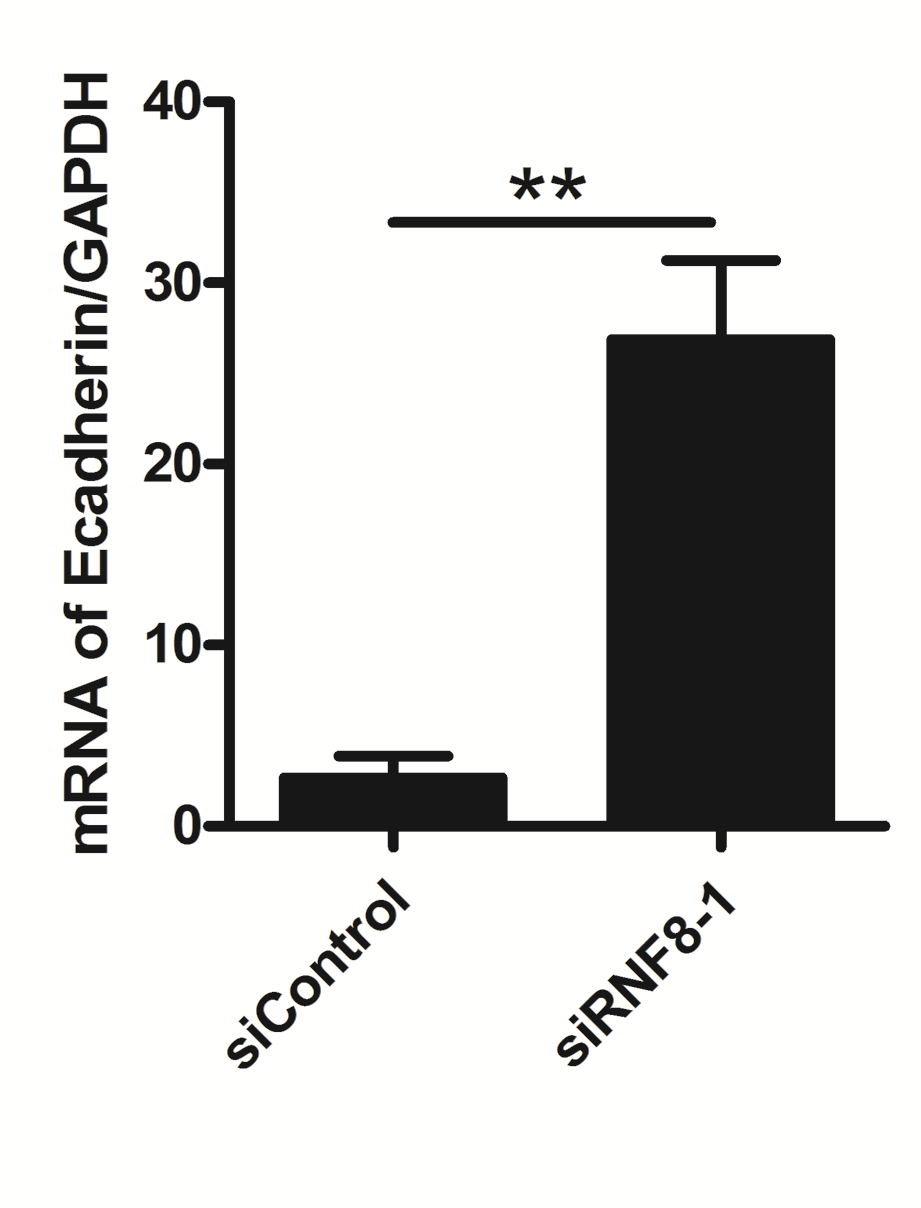


**Figure S1** Real-time PCR analysis of relative E-cadherin mRNA change in RNF8-knockdowned MDA-MB-231 cell line. mRNA of GAPDH was used as a control. Error bars represent mean ± s.d. from three independent experiments; ***p* < 0.01**.**

**
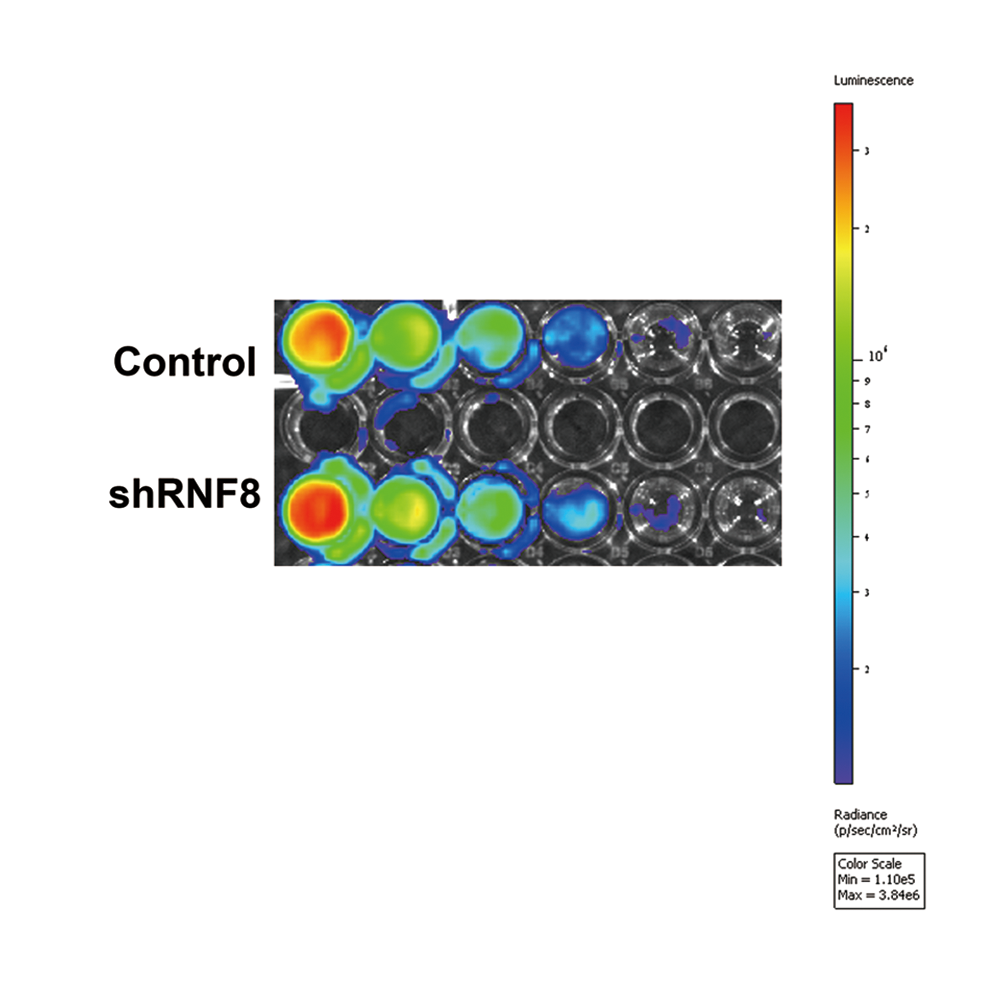
**

Figure S2 Bioluminescence signals of the cells. Before inoculated into the mice, the bioluminescence signals of MDA-MB-231-Luc-siCon or MDA-MB-231-Luc-shRNF8-2 cells were examined by bioluminescence imaging.

**
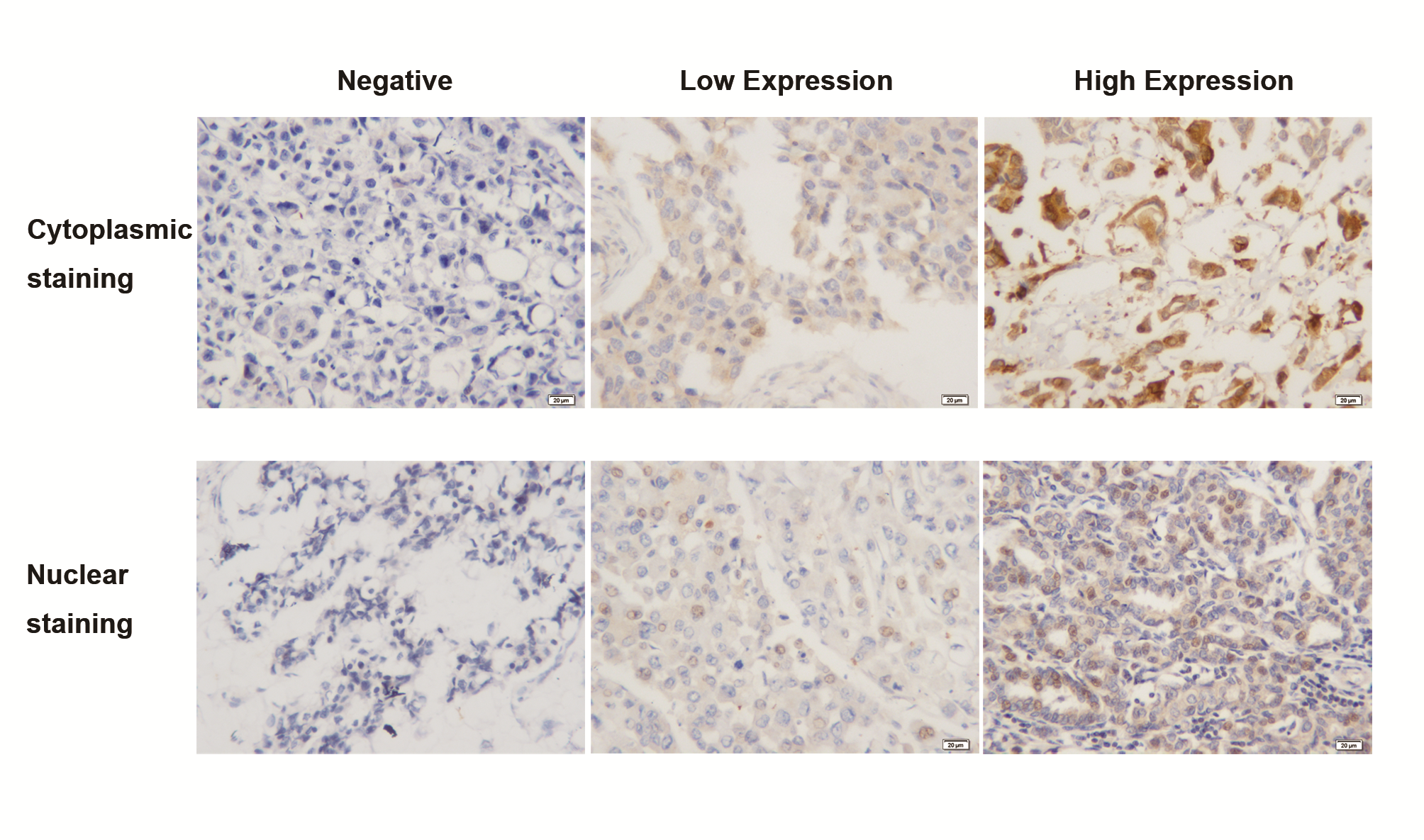
**

**Figure S3** Representative immunostaining pattern in the breast cancer tissues. Images show negative, low and high RNF8 expression respectively.
